# Stereotactic Body Radiotherapy for Lymph Node Oligometastases: Real-World Evidence From 90 Consecutive Patients

**DOI:** 10.3389/fonc.2020.616494

**Published:** 2021-02-05

**Authors:** Petr Burkon, Iveta Selingerova, Marek Slavik, Petr Pospisil, Lukas Bobek, Libor Kominek, Pavel Osmera, Tomas Prochazka, Miroslav Vrzal, Tomas Kazda, Pavel Slampa

**Affiliations:** ^1^ Department of Radiation Oncology, Masaryk Memorial Cancer Institute, Brno, Czechia; ^2^ Department of Radiation Oncology, Faculty of Medicine, Masaryk University, Brno, Czechia; ^3^ Research Center for Applied Molecular Oncology (RECAMO), Masaryk Memorial Cancer Institute, Brno, Czechia; ^4^ Department of Nuclear Medicine and PET Center, Masaryk Memorial Cancer Institute, Brno, Czechia; ^5^ Central European Institute of Technology, Masaryk University, Brno, Czechia

**Keywords:** stereotactic body radiotherapy, lymph node metastases, oligometastases, local therapy, radiotherapy

## Abstract

**Aims:**

To evaluate the efficacy and toxicity of extracranial stereotactic body radiotherapy (SBRT) in the treatment of oligometastatic lymph node involvement in the mediastinum, retroperitoneum, or pelvis, in a consecutive group of patients from real clinical practice outside clinical trials.

**Methods:**

A retrospective analysis of 90 patients with a maximum of four oligometastases and various primary tumors (the most common being colorectal cancers). The endpoints were local control of treated metastases (LC), freedom from widespread dissemination (FFWD), progression-free survival (PFS), overall survival (OS), and freedom from systemic treatment (FFST). Acute and delayed toxicities were also evaluated.

**Results:**

The median follow-up after SBRT was 34.9 months. The LC rate at three and five years was 68.4 and 56.3%, respectively. The observed median FFWD was 14.6 months, with a five-year FFWD rate of 33.7%. The median PFS was 9.4 months; the three-year PFS rate was 19.8%. The median FFST was 14.0 months; the five-year FFST rate was 23.5%. The OS rate at three and five years was 61.8 and 39.3%, respectively. Median OS was 53.1 months. The initial dissemination significantly shortened the time to relapse, death, or activation of systemic treatment—LC (HR 4.8, p < 0.001), FFWD (HR 2.8, p = 0.001), PFS (HR 2.1, p = 0.011), FFST (HR 2.4, p = 0.005), OS (HR 2.2, p = 0.034). Patients classified as having radioresistant tumors noticed significantly higher risk in terms of LC (HR 13.8, p = 0.010), FFWD (HR 3.1, p = 0.006), PFS (HR 3.5, p < 0.001), FFST (HR 3.2, p = 0.003). The multivariable analysis detected statistically significantly worse survival outcomes for initially disseminated patients as well as separately in groups divided according to radiosensitivity. No grade III or IV toxicity was reported.

**Conclusion:**

Our study shows that targeted SBRT is a very effective and low toxic treatment for oligometastatic lymph node involvement. It can delay the indication of cytotoxic chemotherapy and thus improve and maintain patient quality of life. The aim of further studies should focus on identifying patients who benefit most from SBRT, as well as the correct timing and dosage of SBRT in treatment strategy.

## Introduction

Oligometastatic disease (OD), commonly defined as the presence of five or fewer metastatic lesions located in a limited number of organs (1), is now diagnosed more often due to the increased availability of positron emission tomography (PET/CT) scanning, which has become an integral part of the follow-up examination schedule. OD is supposedly an intermediate step between localized and disseminated cancer (2, 3). Local therapies such as surgery, radiofrequency ablation (RFA), cryoablation, or targeted radiotherapy have the potential to achieve local control (LC) with minimal toxicity. In many cases, it can be assumed that if the cancer is in the stage where pathogenic changes leading to dissemination have not yet been promoted, the local treatment of such involvement will lead to a long-term asymptomatic period, or even cure (2–5). Moreover, the possibility of delaying the administration of potentially toxic systemic therapy may significantly affect the quality of life of these patients (6).

Stereotactic body radiotherapy (SBRT) is a non-invasive method of treating localized tumor lesions by applying high doses of ionizing radiation in a small number of fractions. This is possible by employing specially equipped linear accelerators, modern immobilization devices, and imaging methods. SBRT is a short-term treatment that is very well tolerated, non-invasive, and does not require hospitalization or any complicated special preparation, all of which is important, especially in palliative treatment. Compared to standard radiotherapy (RT) techniques, SBRT allows for significantly higher doses to be delivered with less damage to surrounding healthy tissues due to its accuracy (7, 8).

Outstanding local control, improved overall survival, and minimal side effects rank SBRT among the standard treatment methods for localized non-small cell lung cancer and oligometastatic involvement of various sites and different primary tumors (9–11). Most evidence of the use of SBRT in the treatment of oligometastases is mainly related to liver and lung metastases with two-year LC of approximately 80%, the 2 to 3-year disease-free survival (DFS) of approximately 20%, and the 2 to 3-year overall survival (OS) of 25–40% (12–14).

Currently, there is limited data on the use of SBRT in the treatment of lymph node metastases. The number of fractions, and the dose per fraction depend on the location, size, and number of affected nodes. The dose is, of course, limited by the sensitivity of the surrounding structures. Because of their localization, the doses administered in stereotactic irradiation of the affected lymph nodes are lower than in the SBRT of lung or liver lesions. In addition, SBRT is often used repeatedly on a patient.

The aim of this retrospective study is to evaluate the efficacy and toxicity of SBRT in the treatment of 90 consecutive patients with oligometastatic involvement of lymph nodes located in the mediastinum, retroperitoneum, or pelvis. The study was approved by Ethical Board of Masaryk Memorial Cancer Institute (MMCI; approval No. 2020/2802/MOU).

## Patients and Methods

### Patients

The patients screened for eligibility were those who indicated for SBRT in the Masaryk Memorial Cancer Institute between 2011 and 2019. Eligibility criteria included an age of ≥ 18 years, a Karnofsky index of ≥70%, and oligometastatic involvement of lymph nodes located in the mediastinum, retroperitoneum, or pelvis described on a diagnostic CT scan. Before initiating radiotherapy planning, the extent of involvement was confirmed in all patients by PET/CT examination. If additional lesions were found, the SBRT indication was re-evaluated and the patient not meeting the criteria of OD was referred to an oncologist to start systemic treatment. Patients who experienced new metastasis during follow-up after their primary SBRT oligometastases were not re-included in this analysis, even if this metastasis was indicated for another SBRT. In these cases, the evaluation of local control after initial SBRT, time to indicate systemic treatment, and overall survival continued.

### Oligometastatic Disease Classification and Tumor Grouping

OD was classified according to the patient’s history of metastatic disease before diagnosing the treated OD and according to relation to systemic therapy following the system currently presented by the European Society for Radiotherapy and Oncology and the European Organization for Research and Treatment of Cancer (15). The first-time diagnosis of OD is referred to as *de-novo OD*. The term *repeat OD* is used if the patient has a history of oligometastases before the treated OD. Any previous history of polymetastatic disease is referred to as *induced OD*. The development of OD during the systemic treatment-free interval is referred to as *oligorecurrence*. *Oligopersistent* OD is defined as a stable residual disease or partial response occurring during active systemic therapy. The term *oligoprogression* refers to growing or newly developed oligometastases during active systemic therapy. The used system for OD classification is summarized in **Supplementary Table 1**.

Primary tumors were divided into groups considering sensitivity to radiotherapy—*radiosensitive tumors* including breast, head and neck, gynecologic and prostatic (16, 17); *radioresistant tumors* including colorectal, renal, bladder, pancreas, melanoma, sarcoma, and lung tumors (18, 19). In relation to the primary tumor diagnosis irrespective of future OD status, initial disease staging was considered. Initially localized tumors are referred to as *M0*, and patients with initially disseminated disease as *M1* under TNM classification.

### Radiotherapy Technique

A stable, reproducible, and comfortable position of the patient during irradiation was ensured by vacuum-formable mattresses placed freely or in combination with a fixed frame on an linear accelerator couch (20). Until the end of 2015, the combination of vacuum mattresses with the Elekta rigid stereotactic frame (SBF, stereotactic body frame) (21) was used to prevent rotational shifts on the couch. Since January 2016, Frameless fixation of Orfit Industries and CIVCO Medical Solutions has been used in combination with the patient’s position correction in six planes using the Varian PerfectPitch 6DoF couch.

Four-dimensional CT (4DCT) and respiratory gating (management of respiratory movements) during each fraction of irradiation using a linear accelerator with integrated imaging systems and a patient’s respiratory control system were used during treatment planning and subsequent irradiation (22). Gross Tumor Volume (GTV) was defined in 2–3 mm planning CT scans as a lesion visible on CT or CT/MR or CT/PET fusion in all scan sets. This individual GTV from different breath phases was subsequently fused to create an ITV (Internal Target Volume) that included all of the breathing positions. In the case of significant breathing movements, the deep inspiration breath hold technique (DIBH) (23) was used. In this case, the GTV was drawn only at this stage (breath hold). To create the Planning Target Volume (PTV), the GTV was expanded by 3 to 5 mm in all directions (3 mm margin for very well localizable lesions, or when the movements of the tumor were minimal). The prescribed dose was subsequently optimized for this PTV (24).

The risk-adaptive concept was used to prescribe and calculate the radiotherapy dose, where the dose per fraction and the total dose are adjusted to the dose–volume histogram (DVH) of the risk structures (OAR) around the target PTV volume (25, 26). Because of the very close spatial proximity of radiosensitive gastrointestinal structures and spatial instability during repeated fractions (especially in the case of the small intestine), the dose 35 Gy in five fractions was most frequently prescribed, and the median biological equivalent dose (BED_10Gy_) was 60 Gy (in the range of 48–112 Gy).

**Figure 1:**
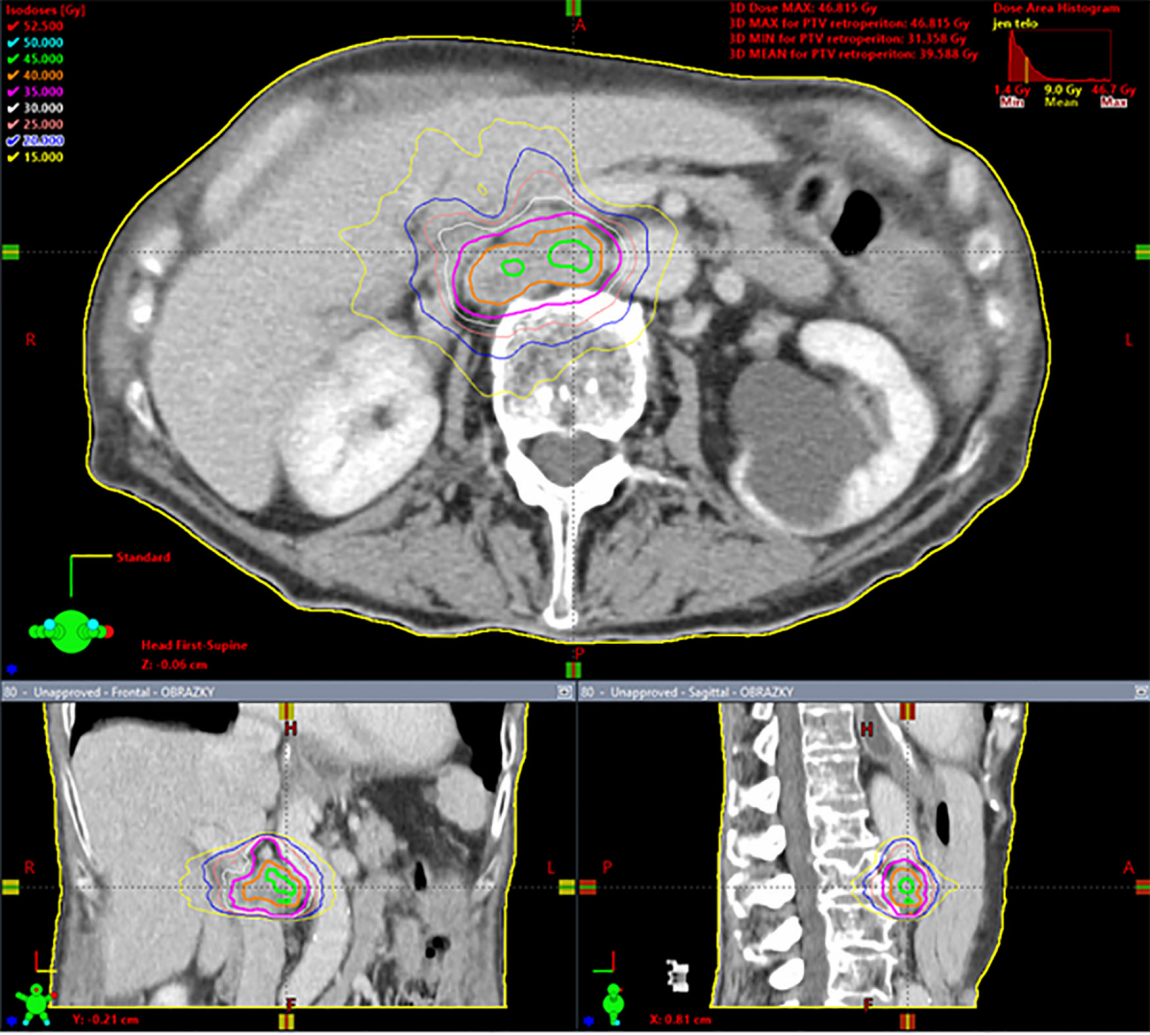
Spinocellular carcinoma of the esophagus in a 63 year-old woman, solitary metastasis in the retroperitoneal lymph node, 35 Gy in five fractions, dose prescription at 80% isodose, PTV = 29.6 cm3, Dmin = 31.4 Gy, Dnear min = 35 Gy, Dmean in PTV = 39.6 Gy, Dnear max = 45.5 Gy, Dmax = 46.8 Gy. Gy, Gray; PTV, planning target volume; Dmin, minimum dose in the PTV; Dnear min, near-minimum dose in the PTV; Dmean in PTV, mean dose in PTV; Dnear max, near-maximum dose in the PTV; Dmax, maximum dose inthe PTV. Dnear min and Dnear max according to ICRU report 83 (29).

Dose calculation was carried out with the Eclipse planning system (Varian Medical Systems, Palo Alto, USA) with the Analytical Anisotropic Algorithm (AAA), enabling heterogeneity correction. Adequate coverage of the target volume was achieved when 98–100% of PTV was covered with 95–100% of the prescribed dose. The best possible treatment plan was also identified by evaluation of the gradient of the radiation dose to the surrounding tissue. This is assessed by 1) the ratio of the isodose volume for which the dose is prescribed to the volume of PTV, 2) the ratio of the volume of 50% isodose to the volume of PTV, and 3) the maximum dose at 2 cm from PTV in all directions. The prescribed dose was applied using the Varian Clinac iX and Varian TrueBeam STX ver. 2.5 linear accelerators equipped with Volumetric Modulated Arc Therapy (VMAT) technology (27) and flattening filter free (FFF) beams, *i.e.*, radiation beams without homogenizing filters. Patient pre-treatment correction was performed online on-board using cone-beam computed tomography (CBCT), which is an integral part of these linear accelerators (28). To ensure patient safety, each RT plan was dosimetrically verified by gamma analysis as part of the standard RT quality assurance process. A typical treatment plan is shown in **Figure 1** and dose constraints are listed in **Supplementary Table 2**.

### Follow-Up, Toxicity, and Effectivity

Patient follow-up during and after SBRT was based on established standards of care in our institution. Follow-up consists of imaging, clinical examination, blood tests, and supplementary examinations according to the irradiated site. The effectiveness of SBRT treatment was monitored in all patients using PET/CT to ensure an accurate comparison with baseline data. If the PET/CT findings were repeatedly negative, more economical contrast computed tomography for the next examination was allowed. The follow-up schedule is as follows: in the first two years after 3–4 months, in the next three years every six months, and then once a year.

Progressive disease was defined according to EORTC-RECIST criteria (30, 31) as a new lesion in the irradiated area or as an increase of ≥20% from the baseline with significant avidity in PET examination compared to threshold activity in the liver. Unclear findings led to the indication of early PET/CT control, biopsy, or surgery. Both acute and late post-radiation changes were evaluated according to the National Cancer Institute’s Common Toxicity Criteria for Adverse Events scale (CTCAE). Acute toxicity occurs during treatment or within the following 90 days. Toxicity evaluation was based on clinical examination and laboratory or imaging data.

### Statistical Analysis and Endpoint Definition

Time-to-event endpoints were outcomes in terms of local control of treated metastases (LC), freedom from widespread dissemination (FFWD), progression-free survival (PFS), overall survival (OS), and freedom from systemic treatment (FFST). All cited events were observed from the date of SBRT termination. LC was determined as the time to progression or recurrence within the PTV. FFWD was defined as the time to distant progression, not amenable to resection or locally ablative therapy. PFS was determined as the time to progression (including local, regional, or distant progression) or death from any cause. OS was defined as the time to death from any cause. FFST was considered as the time to activation of systemic therapy. Patients without the observed event were censored at the date of the last appropriate visit.

Patient and treatment characteristics were described using standard summary statistics, *i.e.*, median and interquartile range (IQR) for continuous variables and frequencies and proportions for categorical variables. SBRT characteristics in patient groups were compared using Fisher’s exact test, the chi-squared test, or the Mann–Whitney test, as appropriate. Survival probabilities were calculated using the Kaplan–Meier method. Survival curves were compared using the log-rank or Gehan–Wilcoxon test, as appropriate. The Cox proportional hazard model was used to calculate hazard ratios. The proportional hazard assumption was verified based on scaled Schoenfeld residuals. Multivariable analysis was performed using backward stepwise selection based on the Akaike information criterion. All statistical analyses were performed employing R version 4.0.2 (32), and a significance level of 0.05.

## Results

### Baseline Characteristics

The basic patient and tumor characteristics are summarized in the left part of **Table 1**.** **A total of 90 patients were analyzed. Men and women were evenly represented, and the median age at diagnosis was 66 years (range 25–80 years). All patients indicated for SBRT were in good general condition, with a Karnofsky index of at least 70%. The most common primary tumors were colorectal cancers (37 patients, 41%). The initial dissemination of the primary tumor was in 19 (21%) patients. Within 12 months of their primary tumor, another 26 (29%) patients developed metastases. In half of the cohort, the first metastases occurred after 12 months.

**Table 1:** Patients’ clinicopathological characteristics (left) and characteristics of oligometastatic disease intended for SBRT (right).

Clinicopathological characteristics of patients (N = 90)		Characteristics of oligometastatic disease intended for SBRT (N = 90)	
**Gender**		**Locality of lesions**	
Female	45 (50%)	Mediastinum	33 (37%)
Male	45 (50%)	Pelvis	17 (19%)
**Age (years)**		Retroperitoneum	40 (44%)
Median (IQR)	66 (55-71)	**Number of lesions**	
Range	25-80	1	60 (67%)
**Karnofsky index**		2	21 (23%)
70	3 (3%)	3	7 (8%)
80	20 (22%)	4	2 (2%)
90	51 (57%)	**History of dissemination**	
100	16 (18%)	De-novo	32 (36%)
**Primary tumor**		Repeat	29 (32%)
Colorectal	37 (41%)	Induced	29 (32%)
Gynecologic/Prostatic	6/3 (10%)	**Relation of OD to systemic therapy**	
Renal/Bladder/Pancreas	9/4/1 (16%)	Oligorecurrence	66 (73%)
Breast	9 (10%)	Oligopersistence	7 (8%)
Lung	10 (11%)	Oligoprogression	17 (19%)
Melanoma/Sarcoma	5/3 (9%)	**Months from last therapy**	
Head and neck	3 (3%)	**(oligorecurrence)**	
**Primary histologic type**		median (IQR)	8 (4–22)
Adenocarcinoma	46 (52%)	range	1–96
GIST	9 (10%)	**Months of ongoing systemic therapy**	
IDC	9 (10%)	**(oligopersistence and oligoprogression)**	
SCC	9 (10%)	median (IQR)	12 (8–23)
Other	16 (18%)	range	4–80
NS	1		
**Initial disease staging**			
M0 (initially localized)	71 (79%)		
M1 (initially disseminated)	19 (21%)		
**Timing of initial dissemination**			
At time of primary tumor	19 (21%)		
Within 12 months of primary tumor	26 (29%)		
More than 12 months after primary tumor	45 (50%)		

SBRT, stereotactic body radiation therapy; N, number; IQR, interquartile range; GIST, gastrointestinal stromal tumor; IDC, invasive ductal carcinoma; SCC, spinocellular carcinoma; NS, non-specified; OD, oligodissemination.

The description characteristics of OD currently intended for SBRT are listed in the right column of **Table 1**. Metastatic lymph nodes were most frequently located in the retroperitoneum (40 patients, 44%) and mediastinum (33 patients, 37%). In the majority of cases, one metastatic node was irradiated (60 patients, 67%). Patients with two lesions (21 patients, 23%), three lesions (seven patients, 8%), and four lesions (four patients, 2%) made up the remaining cases.

In 32 (36%) patients, the treated oligometastases were the first sign of dissemination (*de-novo* OD). The other 58 (64%) patients had been successfully treated in the past for metastatic disease and were currently indicated for SBRT for new oligodissemination. Half of these patients had a history of oligodissemination (repeat OD), and the other half had former multiple metastases (induced OD). Most patients (66 patients, 73%) were not under active systemic therapy at OD diagnosis. The median time without systemic treatment was eight months (range 1–96 months). The other patients had ongoing systemic therapy with a median duration of 12 months (range 4–80 months) at the time of OD diagnosis. No patient in our cohort was treated with concurrent chemotherapy; targeted therapy was also always discontinued at least one week before and one week after SBRT.

The parameters of SBRT treatment and lesion size are given in **Table 2**. The median GTV size was 10.6 cm^3^ (range 0.4–110.2 cm^3^). The tumor lesion size corresponded to the size of PTV (median 27.4 cm^3^; range 3.3–218.4 cm^3^). Patients were most often irradiated in five fractions; the dose was selected according to dose–volume histograms (DVHs) of risk organs surrounding the target PTV volume. More than one-third of the patients were irradiated utilizing schedule 35 Gy in five fractions (35 patients; 39%). A further 29 (32%) patients were irradiated with 30 Gy in five fractions and 11 (12%) patients with 40 Gy in five fractions. The median biological dose equivalent (at *α*/*β* = 10) was 60 Gy (range 48–112 Gy). Only 19 (21%) patients were indicated for systemic treatment immediately after SBRT. According to ICRU recommendations (33), the minimum and maximum doses in 2 and 98% of GTV and PTV volumes were also evaluated.

**Table 2:** SBRT characteristics.

(N = 90)			
**GTV (cm^3^)**		**BED_10_ (Gy)**	
median (IQR)	10.6 (5.2–18.5)	median (IQR)	60 (48–60)
range	0.4-110.2	range	48–112
**PTV (cm^3^)**		**Fractionation**	
median (IQR)	27.4 (14.9–45.2)	3 × 15Gy	1 (1%)
range	3.3-218.4	3 × 9Gy	1 (1%)
**Dmin (Gy)**		5 × 6.5Gy	5 (6%)
median (IQR)	33.5 (29.6–36.9)	5 × 6Gy	29 (32%)
range	23.9-51.9	5 × 7.5Gy	3 (3%)
<30	29 (32%)	5 × 7Gy	35 (39%)
30–37	38 (42%)	5 × 8Gy	11 (12%)
≥37	23 (26%)	5 × 9Gy	4 (4%)
**Dmax (Gy)**		8 × 5Gy	1 (1%)
median (IQR)	36.1 (31.2–41.6)	**Chemo after SBRT**	19 (21%)
range	30.3-58.6	**Acute side effects**	5 (6%)
<37	54 (60%)	**Late side effects**	2 (2%)
≥37	36 (40%)		

SBRT, stereotactic body radiation therapy; GTV, gross tumor volume; IQR, interquartile range; PTV, planning target volume; Dmin, minimum dose; Dmax, maximum dose; BED, biological equivalent dose; Gy, Gray.

### Treatment-Related Toxicity

No grade III or IV toxicity was observed. The most common side effect was mild grade I fatigue, often associated with the need to travel for therapy. Other acute adverse events grade I to II occurred in five patients (6%)—nausea and lumbar pain (SBRT of retroperitoneum), difficulty swallowing, anorexia, and increased mucous production (SBRT of mediastinum) and proctitis (SBRT of the pelvis, pararectally located tumors). Late side effects were observed in only two patients. One patient developed a post-radiation cough associated with post-SBRT infiltrate in the left pulmonary hilus. In the second patient who underwent the SBRT of two nodes in the mediastinum, a traumatic vertebral fracture occurred in the previously irradiated terrain.

### Treatment Outcomes (Time to Event Data)

The median follow-up after SBRT was 34.9 months (95% CI 32.6–43.0). At the time of analysis in August 2020, 58 patients (64%) were still living, and 28 (31%) patients had died from a cancer-related cause. A total of 34 (38%) patients were free of disease and were free of chemotherapy or biological treatment (four patients medicate with hormonal pills). Local recurrence at the irradiation site occurred in 19 (21%) cases, 16 of them with another distant dissemination. These patients were referred for systemic treatment or symptomatic therapy if their general condition worsened. Any progression types (local, regional, or distant) were observed in 66 patients (73%).

**Figure 2:**
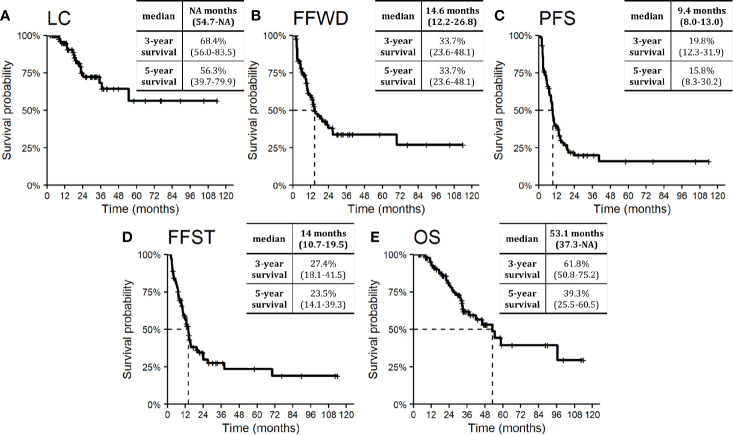
Kaplan–Meier curves for **(A)** local control, **(B)** freedom from widespread dissemination, **(C)** progression-free survival, **(D)** freedom from systematic treatment, and **(E)** overall survival. Dashed lines represent medians. Nested tables include selected characteristics with 95% confidence intervals. NA, Not available.

Median LC was not reached. The probability of absence of local progression at three and five years was 68.4% and 56.3%, respectively (**Figure 2A**). One-third of patients treated with SBRT for OD of lymph nodes did not develop distant relapse that was not amenable to resection or local ablation therapy (*via* SBRT, radio-frequency ablation, or embolization) within five years. The observed median FFWD was 14.6 months (**Figure 2B**).

The median PFS was 9.4 months. Approximately one-fifth of patients (19.8%) were progression-free within three years from SBRT (**Figure 2C**). The necessity of systemic therapy has a significant effect on patient quality of life. In our cohort of patients, the median time to activate systemic therapy was 14.0 months, with a five-year FFST rate of 23.5% (**Figure 2D**). The overall survival rate at three and five years was 61.8 and 39.3%, respectively (**Figure 2E**). Median OS was 53.1 months. During follow-up, 32 (35.6%) patients died, four of whom died without direct relation to cancer.

**Figure 3:**
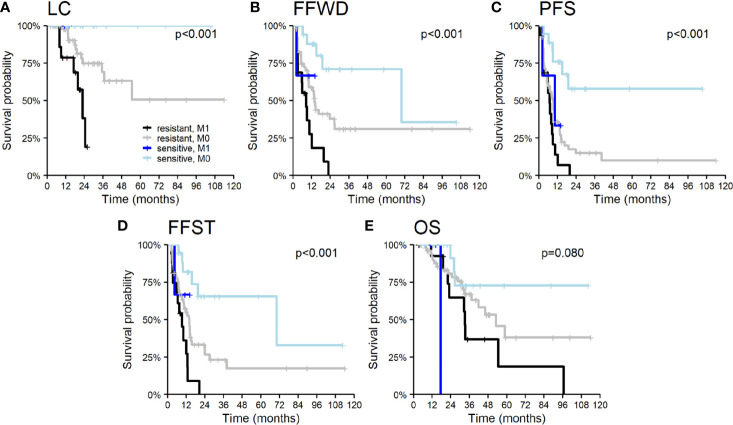
Kaplan–Meier curves according to radiosensitivity of primary tumor and timing of initial dissemination for **(A)** local control, **(B)** freedom from widespread dissemination, **(C)** progression-free survival, **(D)** freedom from systematic treatment, and **(E)** overall survival. The p-values given correspond to the appropriate overall test for the multivariable model.

Patient demographic characteristics, such as age and gender, did not significantly influence survival outcomes except OS. Men had a higher risk of death (HR 2.5, p = 0.012), apparently concerning the unequal distribution of primary tumors. The nature of the primary tumor, together with the initial occurrence of metastases concurrently with the primary tumor, had a major impact on patient prognosis. Tumor aggressiveness expressed by the time to initial dissemination was a negative prognostic factor. The initial dissemination statistically significantly shortened the time to relapse, death, or activation of systemic treatment—LC (HR 4.8, p < 0.001), FFWD (HR 2.8, p = 0.001), PFS (HR 2.1, p = 0.011), FFST (HR 2.4, p = 0.005), OS (HR 2.2, p = 0.034). These results point to higher aggressiveness of the initially disseminated tumors requiring higher radiation doses combined with the maximum possible systemic treatment. Besides, patients classified as having radioresistant tumors had significantly higher risk in terms of LC (HR 13.8, p = 0.010), FFWD (HR 3.1, p = 0.006), PFS (HR 3.5, p < 0.001), FFST (HR 3.2, p = 0.003). Moreover, multivariable analysis detected significantly worse survival outcomes for initially disseminated patients as well as separately in groups according to radiosensitivity (**Figure 3**).

**Figure 4:**
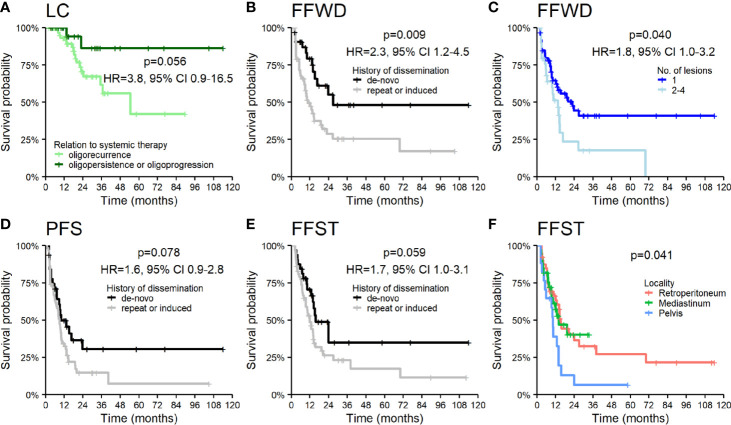
Kaplan–Meier curves for **(A)** local control according to the relation of OD to systemic therapy, **(B)** freedom from widespread dissemination, **(D)** progression-free survival, **(E)** freedom from systematic treatment according to the history of dissemination, **(C)** freedom from widespread dissemination according to the number of lesions, and **(F)** freedom from systematic treatment according to the locality of lesions. Panels include corresponding hazard ratios **(**HR) with 95% confidence intervals (CIs).

The patients under systemic therapy at the diagnosis of oligometastatic disease intended for SBRT had longer time to local progression (HR 3.8, p = 0.056, **Figure 4A**). The patient’s disease history before a diagnosis of OD intended for SBRT has a crucial role in decision making concerning oncological treatment. We analyzed survival outcomes depending on the patients’ previous diagnoses of metastatic disease. Patients with a history of polymetastatic or oligometastatic disease had a higher risk of distant dissemination than patients with *de-novo* OD—FFWD (HR 2.3, p = 0.009, **Figure 4B**). The difference in PFS (HR 1.6, p = 0.078) and FFST (HR 1.7, p = 0.059) did not reach statistical significance (**Figures 4D, E** **)**. Neither LC (p = 0.490) nor OS (p = 0.260) was affected by the patient’s disease history.

The higher number of treated lesions (two to four lesions) increased the risk of distant dissemination not amenable to resection or locally ablative therapy—FFWD (HR 1.8, p = 0.040, **Figure 4C**). The metastases localized in the pelvic area caused a more frequent disease progression, which was related to an earlier indication for systemic therapy (p = 0.041, **Figure 4F**).

**Figure 5:**
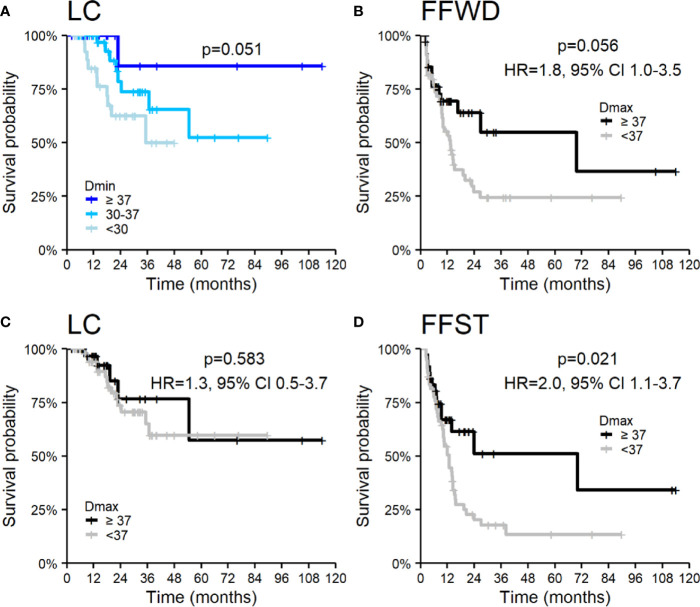
Kaplan–Meier curves according to applied dose expressed by **(A)** Dmin for local control and Dmax **(B–D)** for **(B)** freedom from widespread dissemination, **(C)** local control, and **(D)** freedom from systematic treatment. Panels include corresponding hazard ratios (HR) with 95% confidence intervals (CIs).

SBRT is a local treatment method for cancer diseases. Thus, the essential treatment parameter is the applied dose (Dmin, Dmax, and BED) and its distribution over time (fractionation). Dose and fractionation are primarily related to the location and dimension of lesions. We observed an association of LC with the minimal applied dose on the borderline of significance but not with maximal applied dose. On the contrary, maximal applied dose affected any distant metastases’ appearance and the associated time to activate systemic treatment. The results of the applied dose in relation to survival outcomes are summarized in **Figure 5**, where we categorized Dmin and Dmax into groups using empirically chosen cut-off values. The univariable analysis did not show an association of other SBRT characteristics with the observed survival endpoints.

The complex multivariable analyses with backward elimination (**Table 3**) identified the significance of primary tumor aggressiveness for all treatment endpoints (**Figure 3**), in addition, for LC in combination with relation OD to systemic therapy, and for FFWD and FFST in combination with the lesion locality and number of lesions and (only for FFWD) with Dmax.

**Table 3:** Multivariable analyses for time-to-event endpoints.

	LC p-value HR (95% CI)	FFWD p-value HR (95% CI)	PFS p-value HR (95% CI)	FFST p-value HR (95% CI)
***Primary tumor type*** *Radioresistant vs radiosensitive*	p = 0.01111.2 (1.5,1423)	p = 0.0063.5 (1.4,8.5)	p = 0.0013.4 (1.6,7.1)	p = 0.0033.4 (1.5,7.7)
***Timing of initial dissemination*** *Initial vs. later*	p = 0.0074.1 (1.5,10.8)	p = 0.0032.8 (1.4,5.5)	p = 0.0301.9 (1.1,3.3)	p = 0.0072.5 (1.3,4.7)
***Relation to systemic therapy*** *Oligorecurrence vs. Oligoprogression + Oligopersistence*	p = 0.0613.1 (1.0,15.3)			
***Locality of lesions*** *Mediastinum vs. retroperitoneum* *Pelvis vs. retroperitoneum*		p = 0.0422.7 (1.3,5.8)1.5 (0.7,3.1)		p = 0.0361.6 (0.8,3.2)2.4 (1.2,4.6)
***Number of lesions*** *2–4 vs. 1*		p = 0.0112.2 (1.2,3.9)		p = 0.0541.7 (1.0,3.1)
***Dmax*** *<37 vs. ≥37*		p = 0.0741.9 (0.9,4.1)		

LC, local control; FFWD, freedom from widespread dissemination; PFS, progression-free survival; FFST, freedom from systemic treatment; HR, hazard ratio; CI, confidence interval; OD, oligodissemination.

## Discussion

Oligometastatic involvement of the lymph nodes after treatment of the primary tumor appears in 15–20% of cases and depends on its location and histology (34, 35). Several studies have shown improved patient survival after complete resection of retroperitoneal, intraabdominal, and paraaortic lymphatic relapses (36). However, surgical resection of such involvement is technically challenging, and R0 resection is difficult to achieve (37). Any previous treatment also increases the risk of surgical complications. Such patients are often indicated for systemic treatment—chemotherapy, targeted therapy, or its combination. SBRT, with its potentially ablative doses of radiation, offers an effective alternative to surgery (4). In the present study, local control at 1, 2, 3, 4 and 5 years is approximately 95, 75, 68, 64, and 56%, respectively. Over the same period, PFS is 41, 23, 23, 19, and 19%, and OS is 94, 79, 62, 53, and 39%, respectively.

In Jereczek-Fossa et al. (38), 69 patients underwent the SBRT for oligometastases in lymph nodes (38). The authors report one-year local control of 81.6% and three-year local control of 64.3%. Median PFS was 8.27 months, and three-year PFS was 11.7%. The median OS was 35.4 months. Yeung et al. (39) included 18 patients and reported a one-year local control of 94%, but a two-year local control was only 47%. They also reported a one-year PFS of 39%, a two-year PFS of 17%, a one-year OS of 89%, and a two-year OS of 74%. Despite the high doses (median BED_10_ = 59.5 Gy), the worse local control result could be explained by a high proportion of gastrointestinal tract tumors, especially colorectal cancers, which are considered less sensitive to radiotherapy (37). Loi et al. (40) retrospectively evaluated 87 patients. Their four-year local control and overall survival were high, 79 and 43%, respectively. Franzese et al. (41) recently reported a group of 278 patients with a median follow-up of 15.1 months LC at 1 and 2 years 87.2 and 76.8%, respectively. Better LC was associated with prostate primary tumor, a small tumor volume, oligorecurrence, and BED_10_ ≥75 Gy. One-year OS was 88.4%, and a two-year OS was 73.9%. This study is the most extensive, which includes lymph node oligometastases treated with SBRT. Selected recent SBRT studies evaluating the treatment of lymph node oligometastases are summarized in **Table 4** (6, 38–45).

**Table 4:** Selected recent SBRT studies evaluating the treatment of lymph node oligometastases.

Author, year	No. ofpatients	Primary tumor	Dose	BED_10_	Local control		Overall survival	Toxicity
Franzese, 2020 (41)	278	various	24–54 Gy/ 3–8 fr.	78.8 Gy (37.5–105.6)	87.2% (1 year) 76.8% (2 years)	88.4% (1 year) 73.9% (2 years)		1× gr. 3
Loi, 2018 (40)	91	various	40–48 Gy/ 5–6 fr.	86 Gy (83–113)	79% (4 years)		43% (4 years)	1× acute gr. 3 no late gr. 3
Yeung, 2017 (39)	18	GIT	31–60 Gy/ 4–10 fr.	59.5 Gy (54.8–105)	47% (2 years)		74% (2 years)	no gr. 3 or 4
Jereczek–Fossa, 2017 (42)	94	prostate	median 24 Gy/3 fr.	43 Gy	84% (2 years)		–	no gr. 3 or 4
Wang, 2016 (43)	22	various	median 39 Gy/5 fr.	70 Gy	91% (1 a 3years)		79% (1 year) 43% (3 years)	–
Ost, 2016 (44)	72	prostate			94% (3 a 5 years)		–	no gr. 3 or 4
Jereczek–Fossa, 2014 (38)	69	various	median 24 Gy/3 fr.	43 Gy	64% (3 years)		50% (3 years)	3× acute gr. 3 1× late gr. 4
Bignardi, 2011 (7)	19	various	24–36 Gy/ 1–5 fr.		78% (2 years)		93% (2 years)	no acute gr. 3 1× late gr. 3
Choi, 2009 (45)	30	cervix, corpus	33–45 Gy/ 3 fr.		67% (4 years)		50% (4 years)	6× acute gr. 3 1× late gr. 3

No., number; Gy, Gray; fr., fraction; gr., grade; GIT, gastrointestinal.

The local control achieved in our patients is comparable or better to previously published studies. Yeung et al. reported a lower 3-year LC (47%), explained by a higher number of colorectal cancers and application of SBRT alone, without concurrent systemic treatment (39). The same procedure was used in Jereczek-Fossa et al. with similar results (42). In our study, LC was very good (95 and 68% at 1 and 3 years, respectively) despite the high number of colorectal cancers (41%) and irradiation without concomitant systemic treatment. Besides, in our cohort, the minimum dose in target volume (represented by Dmin in GTV) influences local control. The lower Dmin is always related to the undertreatment of part of the target volume, which is in close proximity to an organ at risk (*e.g.*, duodenum). In our study, LC was worse for patients with Dmin <30 Gy. These observations indicated that the volume of lymph node oligometastases irradiated by a lower dose than 30 Gy in five fractions should be as minimal as reasonably achievable. Nevertheless, the forced undertreatment in the target volume parts near some radiosensitive structure is sometimes inevitable.

Many published studies have also shown better efficacy of ablative doses of radiation (where the biological equivalent of the applied dose exceeds BED_10_ ≥100 Gy) compared to lower doses (46). However, without the most advanced technology, as is MRI guidance, the doses applied to the involved lymph nodes are usually lower. For this reason, the most frequently prescribed fractionation in our population was 35 Gy in five fractions, and the median biological equivalent of BED_10_ was 60 Gy (range 48–112 Gy), corresponding to the gradual development of the learning curve. With appropriate tools and technology, an ablative dose can be given to nodal targets. For example, publications show the feasibility of safely dose-escalating targets such as lymph nodes using MRI guidance (47–49). Even without MRI guidance, Franzese et al. (41) report a higher BED_10_ (median 78.75 Gy). Nevertheless, LC in years 1 and 2 was comparable to our results. A longer follow-up is needed to compare these results further.

In contrast to very good LC, one and three-year PFS was relatively low (41 and 23%), confirming the generally local therapeutic potential of radiotherapy. These values also correspond to the published data; Yeung et al. reported 17% PFS at two years and Jereczek-Fossa 12% PFS at three years. Most of our patients only progressed outside the irradiated area. Whether local progression occurred at the irradiation site, it was almost always associated with multiple dissemination outside the irradiated volume. Reported PFS estimation open discussion about the administration of systemic treatment immediately after solitary lymphatic metastasis is found, or discussion about avoiding indication to SBRT in these cases at all. Conversely, it should be considered that almost a fifth of the patients had been free of signs of disease for five years after the minimal-toxic SBRT and without the need for any other cancer treatment. This number also corresponds to published data after SBRT oligometastasis in the other sites (liver, lung, *etc*.) (7, 50). Approximately 20–25% of oligometastatic patients are free of disease signs for a long period after local treatment, and there is no need to include systemic therapy.

An important observation was the significant difference in all survival parameters in relation to the initial staging of the disease. Specifically, statistically significant differences were found between the group of patients with initially disseminated primary tumor and the group with metastases onset after the completion of primary treatment. Survival differences were observed from both the general disease control point of view (PFS, p = 0.011; FFWD, p = 0.001; OS, p = 0.034; FFST, p = 0.005), and in local control parameters (LC, p < 0.001). No difference in the volume of PTV as well as in the prescribed dose (Dmin, Dmax, BED) was observed between these two groups. These results indicate higher aggressivity of initially disseminated cancer where it is needed maximum possible systemic treatment and a higher radiotherapy dose. Considering that availability of state-of-the-art RT facility equipped by technology for SBRT (or even more advanced technology employing MR guided systems) is still limited, there are specialized centers which provide service to a large region. Especially for extramural patients referred from distant workplaces, it is important to obtain all clinical data about the patient’s disease course irrespective of the possible limited availability of referring medical oncologists.

Future biomarker studies could also identify a subset of patients who have responded to this treatment over an extended period and can benefit from maximum local therapeutical access. These patients could be distinguished from those who would be overtreated by SBRT or even in which the administration of radiotherapy would mean an unnecessary delay in the required chemotherapy (51).

In total, 15 (16.7%) patients of our group progressed beyond the irradiated volume after SBRT again with only oligometastases, which could be re-indicated for local therapy (SBRT, RFA, *etc*.). This allowed further delay of cytotoxic chemotherapy or another type of systemic treatment. Our analysis also assessed the time to multiple progression, which no longer allows the use of local treatment methods (FFWD) and the time to indicate systemic treatment (FFST). Postponing chemotherapy and its side effects thereby improve patients’ quality of life.

Regional relapses or distant oligometastases during follow-up after SBRT should not be a reason to contraindicate other local treatment methods; on the contrary, local treatment methods should be indicated wherever possible to postpone systemic treatment initiation further. In accordance with the fact that all these patients are treated *de facto* with palliative intent, an attempt is made to indicate systemic therapy as late as possible; unless, of course, there is no other clear evidence for immediate administration of chemotherapy or another type of systemic treatment. Further studies will also be required to suggest the optimal timing of SBRT in the treatment of these patients.

The overall survival of patients in our study (unselected cohort treated outside of clinical trials, *i.e.*, real-world evidence) did not differ from the published data. Despite further dissemination, the overall survival rate of these patients was high. Such evidence can help raise the level of evidence of SBRT and its use in routine clinical practice.

The toxicity in our cohort was minimal, with very good local control. SBRT is generally a short, well-tolerated treatment (mostly outpatient) requiring no special training or significant reduction in patients’ quality of life.

We are aware of some limits of our study; first of all, it is necessary to point out the retrospective character of the monitoring, a limited number of patients, and for this reason, it is currently impossible to statistically evaluate efficacy based on tumor type or histology. Also, dose heterogeneity does not allow an optimal treatment strategy to be identified, especially in patients with tumors at higher risk of local failure or early distant spread. The overall survival assessment is biased, of course, on the heterogeneity of further treatment after SBRT. Nevertheless, for practitioners, the OS indicator is an important descriptive characteristic for their indication of this sophisticated radiotherapy method (48).

## Conclusion

Our study has shown that targeted stereotactic radiotherapy, SBRT, is a minimally toxic and very effective local treatment for oligometastatic lymph node involvement. It can delay the use of cytotoxic chemotherapy with minimal patient effort and improve patient quality of life. Less than one-fifth of patients treated in this way survive without signs of disease for an extended period. Identifying patients who benefit most from SBRT in the treatment strategy, as well as its timing and the prescribed dose, should be the subject of further studies.

## Data Availability Statement

The raw data supporting the conclusions of this article will be made available by the authors, without undue reservation.

## Ethics Statement

The studies involving human participants were reviewed and approved by the Ethical Committee, Masaryk Memorial Cancer Institute. The patients/participants provided their written informed consent to participate in this study.

## Author Contributions

PB, TK, MS, PO, and PS conceptualized the study. IS, PB, TK, PP, PO, TP, and MV conducted the data curation. PB, IS, TK, TP, and MV performed the formal analysis. PB, TK, and PS acquired the funding. PB, TK, MS, PP, PO, LB, and LK conducted the investigation. IS, TK, PB, and PP developed the methodology. TK and PB were in charge of the project administration. PS supervised the study. IS, TK, PB, and PP validated the study. PB, IS, TK, and MS wrote the original draft. PB, TK, and IS wrote, reviewed, and edited the manuscript. All authors contributed to the article and approved the submitted version.

## Funding

This work was supported by grants from the Ministry of Health of the Czech Republic AZV 19-00354 and by the Ministry of Health of the Czech Republic—Conceptual development of a research organization (MMCI 00209805).

## Conflict of Interest

The authors declare that the research was conducted in the absence of any commercial or financial relationships that could be construed as a potential conflict of interest.

## Abbreviations

Gy, Gray; PTV, planning target volume; Dmin, minimum dose in the PTV; Dnear min, near-minimum dose in the PTV; Dmean in PTV, mean dose in PTV; Dnear max, near-maximum dose in the PTV; Dmax, maximum dose in the PTV. Dnear min and Dnear max according to ICRU report 83 (51).
