# Supplementary material for: Stereotactic Body Radiotherapy for Lymph Node Oligometastases: Real-World Evidence From 90 Consecutive Patients
Source: Front Oncol. 2021 Feb 5;10:616494. doi: 10.3389/fonc.2020.616494 (PMC7892582; doi:10.3389/fonc.2020.616494)
Supplement: Supplementary file 1 [file Table_1.docx]

**Supplementary Table 1. OD classification** (1)

| **History of dissemination**  Does the patient have a history of metastatic disease before a currently irradiated OD diagnosis? | | |
| --- | --- | --- |
| **De-novo** | **Repeat** | **Induced** |
| Patients without a history of any metastatic disease | Patients with a previous history of oligometastatic disease | Patients with a previous history of polymetastatic disease |
| **Time to development of currently irradiated OD**  Has currently irradiated OD been first diagnosed more than six months after the primary cancer diagnosis?  *This is related only to de-novo OD.* | | |
| **Synchronous** | **Metachronous** |  |
| Early after primary diagnosis (max six months interval between OD and primary cancer diagnosis) | More than six months interval between OD and primary cancer diagnosis |  |
| **Relation to systemic therapy**  Is the patient under systemic therapy at a currently irradiated OD diagnosis? If yes, what is the effect of this systemic treatment? | | |
| **Oligorecurrence** | **Oligoprogression** | **Oligopersistence** |
| Development of OD during the treatment-free interval | Oligoprogressive disease during the active systemic therapy | Stable disease or partial response during the active systemic therapy |
| **Initial disease staging**  Was the primary tumor initially disseminated or localized?  *This is related to the primary tumor diagnosis irrespective of future OD status.* | | |
| **M0** | **M1** |  |
| Initially localized tumor | Initially disseminated disease |  |

**References**

1. Guckenberger M, Lievens Y, Bouma AB, Collette L, Dekker A, DeSouza NM, Dingemans A-MC, Fournier B, Hurkmans C, Lecouvet FE, et al. Characterisation and classification of oligometastatic disease: a European Society for Radiotherapy and Oncology and European Organisation for Research and Treatment of Cancer consensus recommendation. *Lancet Oncol* (2020) **21**:e18–e28. doi:10.1016/S1470-2045(19)30718-1
