# Supplementary material for: Stereotactic Body Radiotherapy for Lymph Node Oligometastases: Real-World Evidence From 90 Consecutive Patients
Source: Front Oncol. 2021 Feb 5;10:616494. doi: 10.3389/fonc.2020.616494 (PMC7892582; doi:10.3389/fonc.2020.616494)
Supplement: Supplementary file 2 [file Table_2.docx]

**Supplementary Table 2. List of dose constrains**(1–3)**.**

| **OAR** | **5 fractions** | | **grade ≥ 3** |
| --- | --- | --- | --- |
| **Lung** | V 20 Gy | 15 % | pneumonitis, lung function |
|  | D 1500 cc | 12.5 Gy |  |
|  | D 1000 cc | 13.5 Gy |  |
| **Bronchi/ trachea **** | D max | 40 Gy | stenosis/ fistula |
|  | D 4 cc | 16.5 Gy |  |
| **Great vessels** | D max | 53 Gy | aneurysm |
|  | D 10 cc | 47 Gy |  |
| **Heart, pericardium** | D max | 38 Gy | pericarditis |
|  | D 15 cc | 32 Gy |  |
| **Esophagus**** | D max | 35 Gy | stenosis/ fistula |
|  | D 5 cc | 19.5 Gy |  |
| **Spinal cord** | D max | 30 Gy | myelitis |
|  | D 0.35 cc | 23 Gy |  |
|  | D 1.2 cc | 14.5 Gy |  |
| **Plexus brachialis** | D max | 30.5 Gy | neuropathy |
|  | D 3 cc | 27 Gy |  |
| **Skin** | D max | 39.5 Gy | ulceration |
|  | D 10 cc | 36.5 Gy |  |
| **Liver** | D 700 cc | 21 Gy | liver function |
|  | D mean | 18 Gy |  |
|  | D 33 % | 24 Gy |  |
|  | D 50 % | 18 Gy |  |
| **Stomach** | D max | 32 Gy | ulceration/ fistula |
|  | D 10 cc | 18 Gy |  |
| **Duodenum**** | D max | 32 Gy | ulceration |
|  | D 5 cc | 18 Gy |  |
|  | D 10 cc | 12.5 Gy |  |
| **Jejunum/ileum**** | D max | 35 Gy | enteritis/ obstruction |
|  | D 5 cc | 19.5 Gy |  |
| **Colon**** | D max | 38 Gy | colitis/fistula |
|  | D 20 cc | 25 Gy |  |
| **Rectum** | D max | 38 Gy | proctitis/ fistula |
|  | D 20 cc | 25 Gy |  |
| **Renal cortex** | D 200 cc | 17.5 Gy | renal function |
| **Renal hilum** | D 66 % | 23 Gy | malignant hypertension |
| **Bladder wall** | D max | 38 Gy | cystitis/ fistula |
|  | D 15 cc | 18.3 Gy |  |
| **Cauda equina** | D max | 32 Gy | neuritis |
|  | D 5 cc | 30 Gy |  |
| **Plexus sacralis** | D max | 32 Gy | neuropathy |
|  | D 5 cc | 30 Gy |  |

Abbreviations: OAR = organ at risk, V = volume, D = dose, Gy = Gray, cc = cubic centimeter

D max is defined as 0.035 cc or less, ** avoid circumferential irradiation.

**References**

1. Benedict SH, Yenice KM, Followill D, Galvin JM, Hinson W, Kavanagh B, Keall P, Lovelock M, Meeks S, Papiez L, et al. Stereotactic body radiation therapy: The report of AAPM Task Group 101. *Med Phys* (2010) **37**:4078–4101. doi:10.1118/1.3438081

2. Lo SS, Teh BS, Lu JJ, Schefter TE eds. *Stereotactic Body Radiation Therapy*. Berlin, Heidelberg: Springer Berlin Heidelberg (2012). doi:10.1007/978-3-642-25605-9

3. de Pooter JA, Romero AM, Wunderink W, Storchi PRM, Heijmen BJM. Automated non-coplanar beam direction optimization improves IMRT in SBRT of liver metastasis. *Radiother Oncol* (2008) **88**:376–381. doi:10.1016/j.radonc.2008.06.001
